# The Knowledge of Neglect: Reflections of Mothers Experiencing Homelessness in Dublin on Social Care Interventions Involving Their Children

**DOI:** 10.1007/s11013-025-09947-y

**Published:** 2025-10-16

**Authors:** Hannah Lucey

**Affiliations:** Department of Social Anthropology, University of Cambridge, Cambridge, United Kingdom

**Keywords:** Care, Homelessness, Addiction, Motherhood, Ireland

## Abstract

This paper examines the reflections on caregiving provided by a group of Irish mothers who were homeless, struggling with addiction, and had lost primary custody of their children. Drawing on Das’s (Slum acts. Polity Press, Cambridge, 2022) discussion of ‘inordinate knowledge’, or knowledge of real-life and morally dubious scenarios that resist attempts at resolution through philosophical reasoning, the paper explores how women in these situations grappled with the realisation of having neglected their children. I demonstrate my interlocutors’ ongoing ethical self-evaluations and continued attempts to make amends for their earlier caregiving lapses, with the limited resources at their disposal. In so doing, I demonstrate how my interlocutors’ yearning towards their children, as revealed through the process of reflexive caregiving action, became entwined with their trajectories through homelessness and addiction in Dublin. At the same time, I probe the limits of anthropologists’ proclivity for contextualising and rationalising unsettling caregiving behaviours. Instead, inspired by my interlocutors’ unflinching self-evaluation, this paper explores the moral quandaries which emerge as a result of care’s polyvalent nature by asking if we can ever reconcile a mother’s attempt to atone for her faltering engagement with caregiving with her child’s experience of neglect.

## Introduction

I’m getting calls from Ellie throughout the day, wondering if it’s ok to meet. She mentions that the social worker has asked her to sign a form for the baby’s passport, but she’s not allowed to bring the child nappies. She mentions this several times, the affront of it. Then she asks what I’ll do with my day. “Maybe walk down the boardwalk by the river”, I say. She replies, “No, I don’t like the boardwalk. I was there for a few weeks in January, you couldn’t move for the fighting”.

“Yeah, I don’t like it either. So maybe I’ll just stay in. Sad old life!”

“No, that’s me”, says Ellie, “Sad old life”.

“You’re trying your best”, I counter.

“No, I don’t think I am. That’s my opinion”.

I am lost for words. “Well, that’s the one that matters”, I offer, eventually.

She agrees.

***

In this paper, I describe the reflections of a group of Irish mothers who, like Ellie, were homeless, struggling with addiction, and distanced from their children because of concerns about their ability to provide care. For many of the women, relinquishing custody involved some element of their consent, as they cooperated with family members and social workers to ensure their children were looked after. This awareness of their mothering limitations was rarely met with indifference or equanimity. Rather, the knowledge of having faltered in their mothering obligations formed an ongoing source of remorse and ethical striving for my interlocutors, as they considered their perceived failures, and pondered over how to make recompense to their children.

Building on Das’s (2022) discussion of ‘inordinate knowledge’—knowledge of real-life scenarios that refuse attempts at resolution through abstract philosophical reasoning—I describe how my interlocutors wrestled with feelings of guilt over their treatment of their children. I detail their attempts to work through this guilt and manage the fallout from their ruminations by either stepping back from or renewing their commitment towards caregiving. The significance of this contribution is twofold. First, on the ground, my interlocutors were often characterised by service providers as people who didn’t or couldn’t care for their children, an interpretation which held devastating implications for the opportunities they were afforded to mother. At its most kindly level, this characterisation was underpinned by a pathologizing logic, an understanding of addiction as an illness that robbed my informants of the capacity to care. At its most cynical level, these assumptions took on a more moralising tone that framed my interlocutors as people who had purposefully chosen drugs over their children. The argument that, instead, my informants continued to reproach themselves and work on redeeming their mothering relationships presents a radical update to these depictions, creating space for an understanding of women who are ‘struggling along’ (Desjarlais, 1997) though homelessness and addiction as mothers who are desperately trying to provide care and whose efforts to mother are dynamic and evolving.

Second, this article nuances anthropological contributions on the experience of mothering while homeless. In general, anthropologists and social scientists have offered a more sympathetic reading than the service providers I grew accustomed to meeting. Many have pointed out that the same structural forces which converge to make certain women vulnerable to experiences like homelessness, such as poverty and racism, also rob them of the resources needed to mother, or indeed, to learn how to mother (Connolly, 2000). For instance, Savage (2022) has argued that caregiving is a capacity which has to be nurtured and invested in like any other skill in order for women to learn how to parent. In contrast, she writes, Irish women exposed to homelessness are repeatedly deprived of opportunities to develop their so-called ‘nurturing capital’, putting them at risk of being inexorably perceived as unable to parent. Social scientists have thus demonstrated that women on the precipice of homelessness are at heightened risk of state surveillance and losing child custody, an outcome with devastating consequences for their mental health and material wellbeing (Bimpson et al., 2020; Carpenter-Song, 2019). These accounts challenge the individualising gaze which holds women in my interlocutors’ position as wholly responsible for their children’s experiences of neglect.

Some anthropologists have gone further and suggested that the experience of mothering amidst poverty, addiction, and state surveillance engenders an ambivalent subjectivity, as women confront their deepening dependence on a state which failed them, and dwindling chances of reunification with their children (Dewey et al., 2018). Some have argued that women living in these conditions simply cannot focus on their children’s wellbeing and must instead prioritise their own survival (Bourgois, 2014; Knight, 2015).

Instead of adopting this rather bleak interpretation, other anthropologists have attempted to stretch our understanding of what it means to provide ‘good’ care. Scholars in this vein have demonstrated the contingent nature of caregiving and the ways in which wider structural forces shape individual practice. In one pre-eminent example, Scheper-Hughes (1992), in her account of mothering in a Brazilian favela, argued that even something as extreme as infanticide may be revealed as a method of rationalising care, allowing its good effects to spread further. Others have made the case that in context, the practice of injecting a friend or family member with heroin can be appreciated as a caring endeavour, a way of ameliorating physical pain (Garcia, 2010; Lancione, 2020). Many anthropologists have thus shied away from labelling instances of ‘bad’ care, instead revealing a contextual, ambiguous, and shifting process. These accounts align with explorations of care as an ethical practice through which people attempt to reach for a good life (Robbins, 2013). Understanding caregiving as an ethical practice necessitates situating individuals within the ‘local moral worlds’ which lend their efforts resonance (Garcia, 2015; Myers, 2015), but also appreciating that people are often scrabbling to materialise caregiving gestures with limited resources at their disposal (Mattingly, 2014).

In general, therefore, anthropologists have demurred from describing examples of caregiving failures and instead highlighted the permutations and innovations which manifest in the field. When I began fieldwork in July 2020, I intended to follow this example. However, I soon realised that the reality was more complicated. During fieldwork, acquaintances and service providers reminded me that some of my interlocutors were persisting in taking drugs despite losing custody or missing visits with their children. My interlocutors, meanwhile, were also keen to confront the possibility that they had been less-than-worthy mothers. As McKearney (2020) has suggested, the reluctance to delve into ethnographic examples of neglect has perhaps deprived us of some important analytical tools, leading scholars to brush over the complicated intentions, emotions and caregiving struggles of those on the ground. In this paper, I take seriously my interlocutors’ insistence that people can fall short in their obligations to care for one another. I argue that my interlocutors’ insistence on taking responsibility for their caregiving mistakes formed an important aspect of their trajectories though motherhood and homelessness, and an important way of registering their ongoing commitment to their children. This paper thus joins an interdisciplinary chorus demonstrating motherhood as a primary locus of meaning for women struggling with mental illness, addiction and homelessness (Bretherton & Mayock, 2021; Nicholson et al., 1998), while adding granular insights into how women's mothering experiences and tribulations were lived on the ground. 

The paper is based on 18 months of ethnographic fieldwork from July 2020 to January 2022, undertaken as part of a doctoral thesis. The setting is the North Inner-City of Dublin, a culturally rich hub in the Irish capital which has become a focal point of homelessness in the wake of the Great Recession in 2008 and subsequent period of stringent austerity (O’Sullivan, 2016). While the breadth of the current housing crisis means that most of those affected were previously stably housed, my interlocutors belonged to a smaller group who had lived through protracted periods of homelessness, alternating between hostels and the streets. Many had grown up in impoverished circumstances and struggled with addiction, mental illness, and disability. More recent research has demonstrated the disproportionate exposure of women in this group to forms of gendered violence, like childhood sexual abuse and domestic violence, relative to both the general population and homeless men (Bretherton & Mayock, 2021). This research also indicates that while women are often treated as single adults within homeless services, many are mothers who have lost custody of their children, but who yearn to be reunited (Mayock et al., 2015; Savage, 2022).

In the hope of learning more about this experience of mothering, I spent time with a loose group of thirty cisgender women. Each had been homeless for over a year, experienced addiction, and lost custody of at least one of their children through interventions by social services or their family members, mostly due to concerns of neglect and drug use. I conducted participant-observation in the waiting room of a GP surgery, a day centre for women who used drugs, and around the Inner-City streets. I also contacted service providers from homelessness charities, child-protection services, and domestic violence agencies, as well as healthcare workers and local government officials, to gain insight into the support structures available to my informants. Due to the outbreak of COVID and the methodological quandaries raised, I conducted virtual ethnography for periods at a time, with informants calling or WhatsApping me when public health restrictions rendered in-person meetings impossible.

Like my interlocutors, I am from Dublin, though a more affluent part of town, a distinction which was not lost on them. In 2016, I qualified as a doctor and worked in this capacity for about two years before starting my studies in social anthropology. During the first wave of the COVID pandemic, I briefly intermitted from my PhD to return to the medical workforce. Considerations about what it means to care well, therefore, as an Irish woman, a doctor and an anthropologist, continually shaped this research.

In what follows, I detail my interlocutors’ deliberations on their struggles with caregiving. In the first section, I reflect on the socio-cultural discourses influencing their sense of being ‘bad’ mothers. In the second section, however, I set these discourses aside, and instead take my interlocutors’ stringent self-evaluations at face-value. Using Das’s discussion of ‘inordinate knowledge’, or knowledge of everyday moral impasses, I demonstrate the pain that the perception of having disappointed their children inspired. Finally, I show how my informants confronted this inordinate knowledge by either withdrawing from or re-engaging with caregiving. My aim throughout is to give voice to my interlocutors’ efforts to live moral lives amongst the hardships and squalor of their everyday circumstances, efforts which I argue are not rendered meaningless simply because they sometimes failed.

## Taking the Blame: Motherhood and Addiction in Ireland

Sometimes, Jessica^1^ would phone me in floods of tears. Many of these conversations fixated on her hostel accommodation: its noise, the meagre cooking facilities, the absence of privacy. Often, however, I got the feeling that Jessica was not really calling me to discuss these indignities. Usually, as these tearful conversations wound round, Jessica would raise the subject of her children. Since becoming homeless in the summer of 2020, Jessica had been separated from her two teenagers, who remained in the care of her ex-partner in what had been the family home. Jessica now only saw them sporadically, whenever they agreed to meet her for a walk or a trip to a café. This seldom happened; however, for although Jessica regularly messaged her children, their responses were terse, unreliable, and infrequent. In the meantime, Jessica was forced to carry on amongst the bedlam of the hostel, with the heartbreak of her absent children hanging over her. In this first section, I examine Jessica’s attempts to understand her current distance from her children. I demonstrate her insistence that she was personally at fault for the separation and explore some of the social and historical forces that may have shaped this damning conclusion.

It took Jessica some time to open up to me about her children. This was in contrast to other revelations which she returned to almost compulsively: the fact that she was ‘illegitimate’, or that she had taken her uncle to court for sexually abusing her as a child, or the perceived cruelty of her ex-partner whom she alleged had emotionally abused her for years. These details were poured over during our phone conversations, with me listening helplessly, wondering if Jessica had been drinking, as she became more and more upset. When COVID restrictions allowed, our discussions took place in person, in the basement of Jessica’s hostel which had been arranged as a kind of sitting room with an itchy couch and some stock images of flowers on the wall. Jessica had recently signed up to an online training course to become a hospital healthcare assistant, although she had not experienced formal education since leaving school at fifteen and was nervous about using a computer. Learning that I was a doctor and could type, she enlisted my help. Once or twice a week, we would find ourselves leaning over her screen, talking through a lecture on the respiratory system or safety in the workplace. During moments of pause, Jessica would insist on fixing me a sandwich, and the conversation would drift back to the events which had led her to the hostel, and sometimes, briefly, to her children.

These discussions usually followed a pattern. Jessica would return to the room with the sandwich (usually ham-and-cheese with crisps on the side—provisions she had bought using her limited funds). As I munched, she would launch into a long, often convoluted, anecdote about the hostel, or the court case involving her uncle, or her ex-partner. She would offer an apology for rambling, saying things like, “My head’s all over the place”, or, “I’m delaying- you can tell”. Finally, she would mention that she had heard nothing from her children. Sometimes she would show me her phone and the trail of one-sided WhatsApp messages. Or, in exasperated tones, she would recount the ways she had tried to support her children over the years. She would recall her pregnancies, for example, using expressive hand gestures to compare the swell of her belly and her tiny frame. She would detail the breakfasts she had prepared in their schooldays (Weetabix were good if they had swimming lessons, she said) or her efforts to magic up the exact Christmas presents they wanted. The specificity of these memories, the ways in which the acts of care that Jessica described had been traced on her body and the food she had prepared (see Carsten, 1997), and the contrast with Jessica’s current isolation from her children always made me feel a rush of sympathy. Whenever I tried to commiserate, however, Jessica bristled. She would shake her head and correct me, informing me that her children’s current distance was in many ways her fault, perhaps most off all because she had started drinking.

These expressions of guilt left me uneasy. By this time, I knew the details of the abuse Jessica had suffered in childhood, not only from her version of events, but from meeting people she had grown up with, and having found newspaper articles about the court case online. I wanted to communicate a sense of solidarity with her individual struggle and the wider cause of tackling homelessness, and hoped that by urging compassion, I might even assist with ‘the hard work of moral repair’ (Myers, 2019, p. 16). I therefore countered that she should not have to take the blame for caregiving mistakes which may have been made, given how these derived from the suffering which had been foisted upon her as a child, through the actions (and inactions) of the adults in her world.

Jessica was not convinced by these arguments, however. She accepted that her uncle’s actions had had a calamitous impact on her life. She also insisted that she had only started drinking in the aftermath of the trial, when for reasons she could not understand, her motivation for tending to her family had ebbed away and she fell into a deep depression; as she said herself, “That shit [depression] is real”. Finally, she recognised that she had been forced to make choices about her children’s upbringing with limited options at her disposal. For instance, Jessica had ultimately agreed to leave the family home, rather than battling to stay and further expose her children to the toxicity of their parents’ relationship. Reflecting on a Christmas that she had spent apart from her family, she once said to me, “I hope my children understand in future that I did it for them, to create some peace. It’s the hardest thing a mother ever has to do”. For many of my interlocutors, the cost of leaving an abusive relationship was homelessness, a decision further complicated by quandaries over how to care for their children. Some decided to leave their children with the relative stability offered by their father rather than bringing them into the dangerous and chaotic system of emergency accommodation, even as they agonised over whether their departure constituted abandonment. Anthropologists have previously explored the ethical dilemmas endured by mothers as they try to make caregiving decisions with a limited well of resources (Parreñas, 2001). Mattingly (2014), for instance, has described the ‘moral tragedy’ faced by African American women who cultivate a ‘superstrong’ personae to advocate more effectively for their sick children, only to desiccate their capacity for interpersonal warmth and play.

Jessica might have recognised these arguments, as a mother whose engagement in caregiving had been curtailed by circumstances beyond her control. At the same time, she appeared to baulk at the suggestion that she could be exculpated from her strained relationship with her children and her reflections on the subject were tinged with self-recrimination. She rebuked herself for locking herself in the bathroom at home to hide her mounting alcohol consumption. She chided herself for lashing out at relatives and even shouting at them on the street. Most of all, she lambasted herself for her absence from her children’s lives during this period and for losing her temper with them at the smallest provocation. While Jessica recognised that this behaviour had been driven by (in her own words) “hurt and pain”, she insisted that the volatility had caused her children deep distress and that, at times, she had failed to live up to a requisite standard of motherhood.

Jessica was not alone in blaming herself for failing her children. Throughout fieldwork, I found my interlocutors received attempts at reassurance in this regard with ambivalence, if not irritation and refusal. There might have been a performative element to these utterances (Carr, 2011). I had witnessed informants perform a kind of self-abasement on other occasions, namely to their children’s social workers, to convince them of their caregiving commitment and capacity for reform. For example, some interlocutors (like Ellie, from the opening vignette) submitted impromptu urine samples for drug screening to the GP, explicitly to try and win over the social worker. It is possible that my interlocutors’ insistence that they were ‘bad’ mothers during our conversations was similarly scripted, an attempt to have somebody recognise them as responsible parents. However, unlike interactions directed at the social worker, I had no influence over the level of access my interlocutors were granted with their children, as they were aware. Moreover, the anguished and effortful manner in which these utterances were made, only after much time was spent getting to know each other and every other topic seemed exhausted, harkened (I felt) to their personal potency, similar to Reece’s (2021) observation that the ways in which our interlocutors share and omit information illuminates their relationship with the past (and with ourselves). I thus began to search for other explanations for my interlocutors’ insistence on self-critique.

The need to take responsibility for caregiving lapses could have been said to flow from locally prominent discourses around motherhood, which coalesced to magnify my interlocutors’ sense of personal fault. The dominant conception of motherhood in Ireland arguably remains that of the altruistic paragon of nurturance (Kennedy, 2004). This image of self-sacrifice holds Marian resonances, in keeping with the fact that all but one of my interlocutors identified as Catholic. Passing on the Catholic faith seemed an important aspect of what my interlocutors considered their mothering role, as evidenced by the frequent discussions I heard about their preparations for their children’s Catholic sacraments, or the fact that Jessica carried prayer cards in her diary. This Catholic experience of motherhood was also steeped in a history of institutionalised shame, perhaps best exemplified by the legacy of the Mother and Baby Homes, the official report into which was published about six months into my fieldwork (Murphy et al., 2021).

The Mother and Baby Homes were religious institutions where single mothers could go to give birth, which flourished after the formation of the Irish State in 1922. They formed part of an interlocking system of carceral social care, funded by the state, and which functioned as an elaborate means of social control in the name of protecting women’s chastity and the moral probity of the nation. Between 1922 and 1998, at least 56,000 women passed through Mother and Baby Homes (Murphy et al., 2021). Once interned there, they were expected to atone for their sins, often by earning their release through menial labour. Most were separated from their children without meaningful consent, while rates of infant mortality were staggeringly high (Murphy et al., 2021). Beyond the cruel treatment meted out to those confined there, the Homes moulded everyday, personal conceptions of morality (Hogan, 2020). Recall that, during our initial meetings, Jessica was fixated on the fact that she was ‘illegitimate’.^2^ She was keenly aware that her mother had been lucky to escape the Homes, once telling me about a schoolteacher who had explained the word ‘bastard’ to her. Historically, therefore, the practice of framing certain women as undeserving of motherhood, and as responsible for the hardships they and their children endured, was structurally embedded in Ireland. I sometimes wondered if my interlocutors willingness to chastise themselves was not redolent of this punitive past.

Building on this, certain discourses around drug use risked ensnaring my interlocutors in a cycle of self-laceration. Addiction was commonly interpreted in my field site through the lens of personal choice, using a neoliberal logic of the rational, autonomous agent (Bourgois & Schonberg, 2009; Burraway, 2018; Singer & Page, 2014), meaning that my interlocutors were commonly assumed to have chosen drugs over their children and intentionally abandoned their maternal obligations. Several anthropologists have demonstrated the ways in which drug use amongst women has been singled out in political discourse as an egregious societal affront, to the extent that in the US, the figure of the mother addicted to crack cocaine was mobilised to justify a racialised war on drugs (Knight, 2015; Singer & Page, 2014). Evidence that service providers in my field site subscribed to similarly damning notions of addiction abounded. Once, while discussing why women in my interlocutors’ position missed visits with their children, for instance, a social worker told me:

You just have to give them [the children] the emotional support, the understanding of addiction... it’s about kind of helping them, I suppose, understand why maybe their parents don’t prioritise access [visits] or significant events for them.

From this perspective, my informants demonstrated a serial disregard for their children, which was fuelled by their addiction. Many of my interlocutors shared similar views, even as they insisted that they loved their children. For instance, Ellie often tried to convince me that drug use was a matter of personal responsibility and that plenty of services existed for people ‘who wanted help’, even as she criticised her child’s social worker for their lack of understanding. This dissonance was interesting, suggesting a view of addiction as a pernicious but wilful lifestyle choice, in the context of which my interlocutors damning self-evaluations began to make sense.

Fidelity to such stringent and unforgiving standards of motherhood and sobriety placed my interlocutors in an existentially perilous position, as they were effectively forced to confront their life trajectories through the prism of personal failure and culpability. Here, I have attempted to sketch the socio-historical discourses around motherhood and addiction which may have exacerbated their sense of guilt. However, and while not wishing to undermine the corrosive effects of such ideals on my interlocutors’ sense of self-worth, it was not just that they were subject to impossible standards. My interlocutors understood these arguments and the need to situate their experiences, and yet, they still claimed that they had sometimes demonstrated caregiving lapses. In what follows, I explore what it means to take those claims seriously.

## Inexorable Knowledge

While my interlocutors agreed that care lapses should be contextualised, they also argued that this information did not completely negate the care-recipient’s experience. In a sense, my informants were making an argument for the fundamentally polyvalent nature of caregiving (see Mol, 2008), and insisting that both the caregiver and recipient’s experiences should be taken into account. For instance, one Monday morning, while visiting Jessica in her hostel, she relayed her disappointment over the fact that her kids had not shown up to a meeting at the weekend. “That’s hard”, she said, “it hurt my feelings. But it’s confusing – it’s so difficult for the kids as well”.

This perception of having faltered in their commitment to their children, even for a moment, formed a kind of internal torture for my interlocutors. To explain this further, I turn to Das’s (2022) discussion of ‘inordinate knowledge’, a concept which builds on the work of philosophers Stanley Cavell and Cora Diamond. Inordinate knowledge, Das explains, refers to knowledge of real-life, morally troubling scenarios which refuse to be resolved through abstract philosophical reasoning. Das gives the example of families in the slums of Delhi who must carry on knowing that their neighbours or kin have engaged in heinous acts of killings, torture, or rape. While some try to enlist the help of the authorities, the outcome of such entreaties can never be sure, meaning that, “faced with such inordinate knowledge, people did what they could in the circumstances that they found themselves in” (Das, 2022, p. 28). The term ‘inordinate knowledge’ thus captures the kinds of knotty social dilemmas and moral impasses that emerge in everyday life as people try to figure out their obligations to one another, where supposedly exceptional occurrences like poverty or political violence have become mundane.

My informants were similarly burdened with inordinate knowledge. Many had kinfolk and partners whom they loved, and yet whom they knew had committed serious crimes. Perhaps even more noxious, however, was the knowledge that they themselves had faltered in their obligations towards their children. The inordinate nature of this knowledge was made clear in those moments when I tried, unsuccessfully, to assuage their sense of guilt and remind them of what they had suffered. Generally unimpressed, my interlocutors countered that contextualising their caregiving lapses did not fully compensate their children for the harms wreaked by their parents’ absence. If Das (2022, p. 20) calls for attention to “the darkness of knowledge that is carried, endured, and worked on in the everyday”, for my informants, this knowledge was that, at times, they had fallen short of their commitment to providing care.

In some cases, this related to specific instances of caregiving lapse. For example, many of my interlocutors felt guilty over their use of drugs during pregnancy. While evidence exists that in some contexts, the long-term impact of antenatal drug use on children has been exaggerated in order to promote punitive drug policies (Knight, 2015; Singer & Page, 2014), nonetheless, drug use during pregnancy poses serious risks to foetal health and is associated with premature birth, low birthweight, and developmental problems. In recognition of these health risks in Ireland, pregnant women deemed at risk of drug use are assigned a so-called drug liaison midwife with specific expertise and are eligible for more frequent ultrasound scans, while women who use heroin are started on methadone treatment to reduce the risk of foetal death and preterm delivery caused by withdrawals (HSE and IOG, 2013). Cognisant of the harms associated with continued drug use during pregnancy, most of my interlocutors ardently desired to cut down; however, many found this ambition very difficult to achieve.

One such lady was Mags, whom I first met at a housing protest early in fieldwork. Over the next few months, I found myself constantly bumping into Mags: outside a shop while she was asking passers-by to help her buy formula milk, or queuing for a soup run. During each of these impromptu meetings, I observed Mags doting on her baby, producing yoghurts, biscuits and toys from the undercarriage of her pram. In conversation, she described the child as ‘the reason I get up in the morning’, and credited him as the motivation for stopping using drugs. However, Mags also informed me that she had smoked heroin and crack right up until the moment her contractions began, in an alleyway beside the hospital. During another conversation, as we sat on a street bench, she gestured towards the apparently bonny child, and asked me, eyes wide, how she could have jeopardised the baby’s health and future. Afterall, she said, it was still too early to know what the effects of her drug use would be. It was all I could do to make a sympathetic face, while Mags shook her head, shuddering.

As my fieldwork progressed, I learned that these struggles to achieve abstinence took place in a highly specific context. Pregnancy often formed an acutely destabilising experience for women in my interlocutors’ position; as one drug liaison midwife I spoke to explained:

Pregnancy is often spoken about as an ‘incentive for change’, but in my experience, it’s not as simple or rosy as that. Often, they’re crisis pregnancies, and they introduce stress and chaos, even for a person who’s trying to stop [using drugs].

As this quote alludes to, many of my interlocutors’ pregnancies were unplanned, and sometimes a consequence of sexual assault. One informant told me that although she still loved her children thus conceived, the circumstances surrounding her pregnancies had hurt her engagement with motherhood (Knight, 2015), as well as her capacity to stop using drugs.

Homelessness was heaped on top of these anxieties. Few specialised accommodation services for women who are homeless and pregnant exist in Dublin, and even fewer where heavy drug use is concerned. Pregnant women can find themselves relying on the hostels to avoid sleeping rough, with infrastructure vastly unsuitable for their needs. Jessica once recalled watching Ellie struggling up the stairs of their hostel, a shabbily repurposed, four-storey Georgian building, when she was nearly due. Another interlocutor complained about being allocated a top bunk bed when she was 4 months pregnant. I learned from a social worker from one of the maternity hospitals that women in my interlocutors’ position were often returned to so-called 'low-threshold’ hostels after giving birth, settings where drug use was commonplace, even though as women who had just lost custody of their babies, they were particularly vulnerable to relapse.

Most excruciatingly, my interlocutors could go the duration of their pregnancies not knowing if they would be allowed to keep their children. Mags remembered calling her aunt from the delivery suite, weeping, “What will I do if they take [the child]?”. This uncertainty overshadowed my informants’ pregnancies and further undermined their efforts to curb drug use. While Knight (2015) has argued that the privations associated with being homeless and addicted to drugs stripped her interlocutors in San Francisco of the luxury of caring for their unborn children, I found it was the stress inherent to being pregnant while homeless that pushed some of my interlocutors towards substance use. For some, it was all they could manage to reduce the drugs they were using, rather than ceasing entirely.

The above outlines the context for my interlocutors’ faltering attempts to reduce their drug use, a context they were brutally aware of. Yet, this knowledge barely dented their feelings of guilt once their children were born. Although cerebrally they understood why their drug use had continued, they found the reality of having continued to use drugs difficult to reconcile, a kind of ‘inordinate knowledge’ that weighed heavily on their sense of themselves as mothers who cared.

In other cases, ‘inordinate knowledge’ referred to less specific moments of caregiving lapse. Many interlocutors berated themselves for allowing their personal experiences of suffering to contaminate their children’s lives. To explain this, I turn to Genevieve, whom I first met in the GP’s waiting room and over time, became acquainted with the details of her past. Genevieve had been removed from her parents’ care as a child and placed in a religious care institution, variously described in official documents as a Mother and Baby Home. There, she had been sexually abused by a member of staff, before being transferred to a youth hostel. At some point, Genevieve started using drugs and was sent to prison for a crime she swore she didn’t commit. To her shock, and for reasons that never became clear, by the end of my fieldwork, she had been incarcerated once again.

Just preceding her re-arrest, Genevieve was given an apartment through a homelessness charity. At this point, she was heavily pregnant but had mixed feelings about the baby’s arrival. Genevieve already had a child in foster care. Although she had agreed to rescind custody of the new baby when the time came, the prospect of another separation, and of having her care skills audited at every access visit was filling her with dread. More distressing still, she told me, as we were sitting in her new flat looking through photo albums she had made of her older daughter, was the knowledge that she hadn’t protected her children from the suffering which had overshadowed her life, and that she had allowed her misery to seep into theirs. Genevieve was overcome by this thought and began to cry, wondering aloud if her children would be better off if she just disappeared. We worked to steer the conversation into less distressing terrain; I asked Genevieve about school, and she offered me another cup of coffee. Not long after, her partner returned home, and at Genevieve’s gentle request, I left.

A special edition of *Ethnos* considers the question of ‘contagion’ in kinship (Meinert & Grøn, 2020), pointing out that as well as biogenetic substance, lived-experiences are passed down through generations, through idioms of haunting (Meinert, 2020) or experiences of confused, adultified, and interrupted childhood (Han & Brandel, 2020). In part, this work explores how legacies of suffering and exploitation come to be inherited, even by descendants who were not alive at the time of the initial injury. Similar questions of generational ‘contagion’ were uppermost in the minds of the service providers I spoke to, who lamented that they were now seeing the children and grandchildren of former service users walk through their doors. My interlocutors also seemed concerned with contagion, although they did not frame this in terms of social or structural issues. Instead, using different phraseology, they described a sense of being stalked by suffering all their lives, and their guilt at failing to lift this curse from their children. Jessica, for example, berated herself for not only becoming lost in her “hurt and pain” after the court case, but for allowing this “hurt” to reach into her children’s lives as well.

These self-admonishments often seemed rooted in my interlocutors’ early exposure to sexual violence and a perhaps unreasonable expectation on themselves to inoculate their children from the past. However, to reduce my interlocutors’ reflections to a performance or an internalisation of unrealistic standards would be to underplay the serious natures of the quandaries they faced. My interlocutors could not but help asking what they could have done to spare their children from pain. In so doing, they forced me to confront some truths about caregiving. For even if imperfect care can be explained, they seemed to argue, and even if these rationalisations are important to reconfiguring our evaluations of the caregiver, they might not be enough to comfort the recipient nor assuage their abiding sense of neglect.

## Trying Again

More than just the contents of inordinate knowledge, Das urges us to attend to the ways that people live with such knowledge and build meaningful lives amongst the irreconcilable conundrums presented by the everyday. In the following section, I attempt to show how my interlocutors shouldered the inordinate knowledge of having faltered in their caregiving endeavours and how accepting this burden shaped their relationships with their children.

Das suggests that through conjuring up variegated acts of care, her informants find the courage to overcome their awareness of other people’s transgressions and persevere with their everyday lives. However, my interlocutors’ handling of inordinate knowledge was different because the knowledge they were burdened with pertained to themselves. For my respondents, rather than setting aside inordinate knowledge, reknitting their social worlds required its forthright acknowledgement, including the personal culpability contained therein. This willingness to recognise personal fault was understood as itself an act of care, through which my interlocutors registered their concern for their children and assumed at least partial responsibility for the care lapses the younger generation had suffered. When Jessica argued that she should not have lost sight of her children’s needs, or Genevieve lambasted herself for passing on a legacy of wretchedness, both mothers were attempting to confront the past, reaffirm the centrality of their children’s wellbeing, and identify aspects of their own caregiving conduct which required improvement.

It was not enough to rhetorically assume blame. Instead, my interlocutors worked to consolidate this gesture by materialising acts of care. Acknowledging inordinate knowledge was not the same as asserting a static view of themselves as inherently ‘bad’ or incapable parents, therefore. Rather, accepting responsibility became the first step in a process of trying again. For some, reinvigorated caregiving efforts were directed beyond their kin. Jessica’s attempt to train as a healthcare assistant could be viewed in this light, particularly given that she dreamed of working in a children’s hospital. As another example, whenever Mags spotted a pregnant woman out ‘tapping’ (a colloquialism for begging), she would urge them to enter addiction treatment, using her own story as inspiration and letting them peer into her buggy to visualise what they stood to lose. Rather than shying away from her previous caregiving lapses, Mags was attempting to work through this knowledge, reaching out to her peers and helping them to avoid similar pitfalls.

More often, my interlocutors’ redoubled caregiving efforts focussed on their own children. Some tried to get pregnant again, in the hope that with a new child, they would be able to rewrite the past. This response elicited a lot of concern and frustration from service providers; one doctor told me that he almost dreaded this news from his patients who were homeless, as pregnancy seemed to feed the pernicious cycle of anxiety, escalating drug use, and social work intervention. For others, redoubled caring efforts focussed on their already distanced children. After periods of silence, for instance, Ellie would contact the social worker and insist that she wanted to be re-involved in her child’s life. Others tried to rekindle their relationships with adult children, in the hope that it was not too late to transform the nature of their bond.

This was true of Christy and Nadine, whom I met after they had been given an apartment through a housing charity, having spent 5 years alternating between hostels and the streets. Christy and Nadine’s experience of homelessness had been precipitated by losing custody of their child, an intervention which they both insisted had been unjustified. Regardless, they lambasted themselves for what had happened next. “Like a thick”, Christy said, he had, “buried [his] head in the sand”, and started injecting heroin, while Nadine concurred that she had lost herself in drug use, a tale of existential deterioration in the wake of child removal which I became accustomed to over the course of my fieldwork (see Carpenter-Song, 2019). What struck me more on this occasion was how the couple berated themselves for succumbing to despair, rather than advocating for themselves as parents. The realisation that in dissolving into drug use, they had confirmed the social workers’ worst impression of their caregiving capacity and reinforced their daughter’s absence from their lives formed a kind of inordinate knowledge, a calcified source of self-loathing and guilt.

Now, 5 years later, Christy and Nadine were trying to acclimatise to their new apartment. Almost as soon as they moved in, they began to use this resource to reknit their ties with kin, which had been taxed and frayed by the experience of homelessness. Christy had another daughter, it transpired, who was herself a mother to a small child and struggling to find accommodation. Christy was tormented by his absence during his daughter’s childhood, and so, when the couple were given the apartment, he invited her to live with them. When I visited, Christy’s adult daughter had been given the bedroom to sleep in, while Christy and Nadine had taken the couch. Photos of the toddler were stuck to the fridge, while coloured scribbles were faintly visible around the skirting boards. Though not biologically related to the arrivals, Nadine was very happy with the arrangement and told me that the child had started to refer to her as ‘Nan’.

Visiting Nadine and Christy was to witness the processual aspects of kinship, and how these were engendered by the sharing of domestic space (Carsten, 1997). In the corner, I could see a heap of clean laundry, waiting to be folded, while the couple vented with mixed exasperation, amusement, and pride about scrubbing the toddler’s writing off the walls. Furthermore, Christy and Nadine were clearly using their newfound resource to try and atone for past caregiving mistakes. By sharing the home and then repeatedly enacting domestic caregiving gestures, they were able to offer a young mother the support which had been absent for them as parents, as well as demonstrate their renewed commitment to family, and recoup some of the caregiving experiences which had been thwarted by the removal of their child. Christy and Nadine did not deny having been imperfect parents. However, they were trying to atone for this knowledge by re-engaging with care.

In the above section, I have detailed different methods of materialising care: through imparting advice to strangers, or partaking in mundane household chores. I would argue that a kind of ‘internal consistency’ (Das, 2022, p. 130) links these gestures, as my interlocutors’ reflections on their caregiving lapses inspired the kinds of care they decided to manifest. Mags used the living, breathing evidence of her infant child to convince other women not to lose themselves (as she had once done) to drugs. Nadine and Christy attempted to assert their presence through sharing their home, where once they had been absent. Each of these acts was connected by an internal logic of response, as my interlocutors made the inordinate knowledge of caregiving lapse explicit so that they could intervene within and against the evidence of having previously faltered. While anthropologists have previously argued that people’s efforts to live moral lives rely on their building intimate connections in a way that can be recognised locally as ‘good’ (Myers, 2015), I would add that these efforts often incorporate the knowledge of having previously stumbled. By responding to the ‘inordinate knowledge’ of having been imperfect mothers, my interlocutors materialised the hope of different maternal relations in the future or, at the very least, a better future for their children than past for themselves.

However, my interlocutors did not always appreciate my positive evaluation of their caregiving efforts. After all, if they were still using drugs and apart from their children, could it really be said that they were trying, or that their incipient, stalling efforts held value to anyone beyond themselves? Their scepticism formed a cruel reminder of the dark underbelly of inordinate knowledge, which although offering a source of motivation, also sowed a kind of canker, choking my interlocutors’ attempts at cultivating a relationship with their children. Many, like Genevieve, worried if they were unworthy of motherhood, and if it would be better for their children if they disengaged. Others used drugs to obliterate their guilt, further demonstrating the pernicious feedback loop that drugs represented, as women blamed themselves for allowing their addiction to interfere with their mothering, and then used drugs to try and soothe the ensuing shame.

Thus, the knowledge that their commitment to caregiving had wavered often proved a kind of torture for my interlocutors, seeming to confirm their sense of worthlessness and clouding their willingness to acknowledge the structural factors constraining their capacity to provide care. Instead, inordinate knowledge encouraged my interlocutors to assume all the blame for their children’s experiences of neglect, damning themselves and eating away at their caregiving capacity and resolve.

## Conclusion

This paper has aimed to explore my interlocutors’ reflections on their caregiving limitations. It has focussed on the complexities of women’s own moral assessments and their everyday attempts to navigate the past, their drug use, and somehow forge liveable lives. Through this, I have shown that women’s caregiving lapses were a source of tremendous self-laceration, to the extent that my interlocutors’ ruminations sometimes seemed to feed into insidious narratives around maternal obligation and the ‘choice’ to do drugs. At the same time, their unwillingness to shy away from the possibility that there was some element of themselves in the experience of caregiving lapse was revelatory about the ethical quandaries which congeal around the subject of care, especially for those who live such precarious lives. While my interlocutors understood that the experience of poverty and exclusion had at times eviscerated their capacity to respond in mutually satisfactory ways to their loved ones, they also argued that these contextual factors did not undo the suffering which being absent had caused their children.

Anthropologists have at times appeared to demur from discussing the ways in which structural suffering injures our ability to respond to other people’s needs, perhaps fearful of lending legitimacy to accounts of anomie which distract from those innovative forms of care which do emerge within conditions of privation. In so doing, however, they have potentially underplayed the challenges of caregiving, and especially for those experiencing the full wrath of society’s disdain. The accounts of caregiving I have presented capture moments of caregiving ambivalence, refusal, and self-destructiveness. Acknowledging these moments of faltering as faltering (Lucey, 2025), rather than relative and contingent successes, ensures that we do not inoculate ourselves from the quandaries that most afflict and distress homeless mothers as they go about tackling their caregiving labours within the everyday.

Further, allowing for the possibility of caregiving lapse means that homeless women’s attempts at re-engagement can be acknowledged, as they tried to re-immerse themselves in the obligation (and moral worth) that comes from caregiving. This is important because the moments of lapse I witnessed were often just that, fleeting moments, which women were able to overcome. Just because my interlocutors faltered wasn’t the same as saying they didn’t or couldn’t care for their children; in fact, caregiving offered both the means and motivation for pushing through the knowledge of failure. In a sense, my interlocutors were continually making the claim that although mistakes had been made, this did not and should not disqualify them from ever taking up an active mothering role. Their efforts to work through the inordinate knowledge were redemptive, in suggesting that the frayed threads of their relationships could be knitted back together, with effort and with time.

## Data Availability

No datasets were generated or analysed during the current study.

## Footnotes

1 I have anonymised my interlocutors, including details of their children.

2 The legal basis for ‘illegitimacy’ in Ireland was abolished in 1987.
